# RTL1 promotes melanoma proliferation by regulating Wnt/β-catenin signalling

**DOI:** 10.18632/oncotarget.22523

**Published:** 2017-11-20

**Authors:** Guobiao Fan, Dan Ye, Songcheng Zhu, Jiajie Xi, Xudong Guo, Jing Qiao, Yukang Wu, Wenwen Jia, Guiying Wang, Guohuang Fan, Jiuhong Kang

**Affiliations:** ^1^ Clinical and Translational Research Center of Shanghai First Maternity and Infant Health Hospital, Shanghai Key Laboratory of Signalling and Disease Research, Collaborative Innovation Center for Brain Science, School of Life Science and Technology, Tongji University, Shanghai 200092, China; ^2^ Skin and Cosmetic Research Department, Shanghai Skin Disease Hospital, Tongji University, Shanghai 200443, China

**Keywords:** melanoma, proliferation, retrotransposon-like 1, β-Catenin, Wnt signalling

## Abstract

Cutaneous melanoma is a highly malignant and metastatic skin cancer with high mortality. However, its underlying mechanisms remain largely unclear. Here, we found that retrotransposon-like 1 (RTL1) is highly enriched in melanoma tissue, especially in early and horizontal growth tissues. Knockdown of RTL1 in melanoma cells resulted in cell proliferation suppression; cell cycle arrest at G1 phase; and down-regulation of E2F1, CYCLIN D1, cyclin-dependent kinase 6 (CDK6) and c-MYC. Moreover, overexpression of RTL1 in melanoma cells accelerated cell proliferation, promoted passage of the cell cycle beyond G1 phase, and increased the expression of cell cycle related genes. Mechanistically, we found that knockdown of RTL1 inhibited the Wnt/β-Catenin pathway by regulating the expression of genes specifically involved in β-CATENIN stabilization. Furthermore, the overexpression and knockdown of β-CATENIN rescued the effects of RTL1 on melanoma cell proliferation and the cell cycle. These findings were also confirmed via tumour xenografts in nude mice. Together, our results demonstrated that RTL1 promotes melanoma cell proliferation by regulating the Wnt/β-Catenin signalling pathway.

## INTRODUCTION

Cutaneous melanoma is a highly aggressive skin cancer and is responsible for over 80% of all deaths among skin cancer patients. The mortality of melanoma increased nearly 165% worldwide from the 1950s to 2000 [1], and the incidence has increased continuously over the past decade [2–4]. Because malignant melanoma is not sensitive to chemotherapy or radiotherapy, both of which are deleterious to a patient's immune system [5–7], surgery is the most effective treatment for early melanoma with a 5-year survival rate of 89-96%. However, the time window for surgery is very narrow, and many patients miss the chance to undergo surgery, owing to metastasis. Recently, gene therapy and immunotherapy have emerged as potential therapeutic approaches for melanoma, although the underlying mechanisms remain to be elucidated.

Retrotransposon-like 1 (RTL1) is a Ty3/Gypsy transposon located in the conserved *Dlk1-Dio3* imprinted region [8]. RTL1 plays an important role in maintaining the normal placental barrier, and it affects the growth of embryonic blood vessels when it is abnormally expressed, thus causing embryonic deformity or embryo deaths [9]. Interestingly, RTL1 has recently been reported to be associated with the development of several cancers. The methylation level of the RTL1 promoter is significantly lower in lung cancer tissues than in normal lung tissues. Further, the expression of microRNAs in the exon of RTL1 is increased [10], and RTL1 is highly expressed in mouse liver cancer induced by the Sleeping Beauty (SB) gene [11]. Because RTL1 is the only consistently altered gene detected in all SB-induced tumours with *Dlk1-Dio3* integrations, it has been suggested that RTL1 activation is a driver of liver cancer [12]. Recently, it has been reported that the expression level of RTL1 was significantly increased in the squamous cell carcinoma of head and neck, but the function of RTL1 was not described [13]. Thus, little is known about whether RTL1 is involved in the development of melanoma.

It has been reported that the Wnt/β-Catenin pathway is critical for embryonic development, haematopoietic function, and wound repair [14–18]. Numerous studies have shown that the Wnt/β-Catenin pathway is aberrantly activated in melanoma cells, animal models and patient tissues [19–22]. β-Catenin accumulates in the nucleus and activates proliferation-related genes in melanoma cells, thus leading to the deterioration of melanoma [23–26]. However, β-Catenin may excessively accumulate in the cytoplasm, translocate into the nucleus, and promote the expression of c-MYC [27], CYCLIN D1 [28], and AXIN2 [14]. Additionally, in the cytoplasm, GSK3β regulates β-Catenin by regulating its phosphorylation and degradation, whereas in the nucleus, β-Catenin is regulated by TRIM33 [29]. Moreover, the Wnt/β-Catenin pathway has been reported not only to be a predisposing factor of melanoma, but also to lead to the progression and worsening of melanoma [30].

In the present study, we observed that RTL1 was highly expressed in melanoma tissues, and was critically involved in the proliferation of melanoma cells *in vitro* and tumour growth *in vivo*. Further, we found that RTL1 activates the Wnt/β-Catenin signalling pathway by regulating the expression of β-Catenin stabilizing proteins, thus promoting cell cycle progression in melanoma cells.

## RESULTS

### RTL1 is highly expressed in human skin melanoma

**Figure 1:**
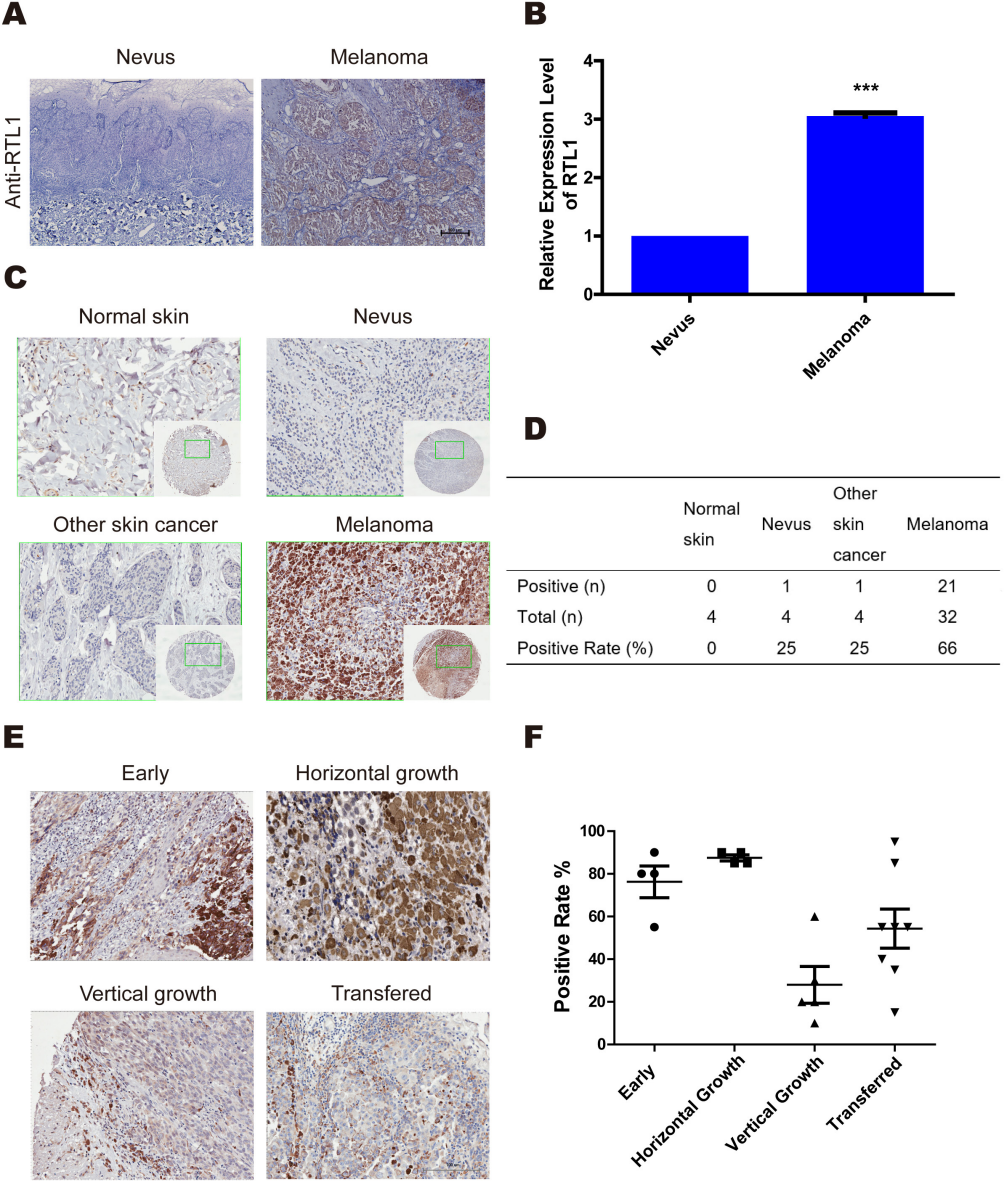
RTL1 is highly expressed in human skin melanoma **(A)** Immunohistochemical analysis of the expression of RTL1 in human melanoma and naive tissues; three tissues were examined; **(B)** The expression of RTL1 was analysed using RT-PCR, as detailed in the Methods, ^***^ p<0.001; **(C, D)** Tissue array analysis expression levels of RTL1 in control human skin, nevus, melanoma, and other skin cancer. The expression of RTL1 was higher than that in other tissue; **(E, F)** The RTL1 staining positive rates in the early phase, horizontal growth, vertical growth, and metastasis states were compared, and the RTL1 positive rate was higher in the early phase, horizontal growth states.

To explore the role of RTL1 in melanoma, we examined the expression of RTL1 in the nevus and melanoma, and found that RTL1 was highly expressed in melanoma tissues. Quantitative real-time PCR (QPCR) analysis confirmed the significantly higher expression of RTL1 in melanoma tissues than in nevus tissues (Figure 1A, 1B). Tissue array analysis was also used to detect the expression of RTL1 in normal skin, nevus, cutaneous squamous cell carcinoma, basal cell carcinoma, and melanoma (Table 1). These findings showed that RTL1 was more frequently upregulated in melanoma than in other tissues (Figure 1C, 1D). The results of the positive rate in different melanoma phases showed that the expression of RTL1 in early and horizontal growth melanoma was higher than that in the vertical growth and metastasis states (Figure 1E, 1F). These results indicated a significant role of RTL1 in early stages of melanoma.

**Table 1:** Clinical and pathologic characteristics of patients with melanoma

Characteristics	Number	RTL1 Score	*P* value
Sex			
Male	16	1.59	0.13
Female	16	3.03	
Age (yrs)			
<60	22	1.68	0.07
>60	10	3.48	
Lymph node metastasis			
Negative	20	2.29	0.95
Positive	12	2.35	

A *p* value greater than 0.05 indicates no significance difference. The 32 patients are divided into two groups according to their sex, age, and metastasis status. The numbers of patients in each group are shown in the column.

### RTL1 promotes the proliferation of melanoma cells

**Figure 2:**
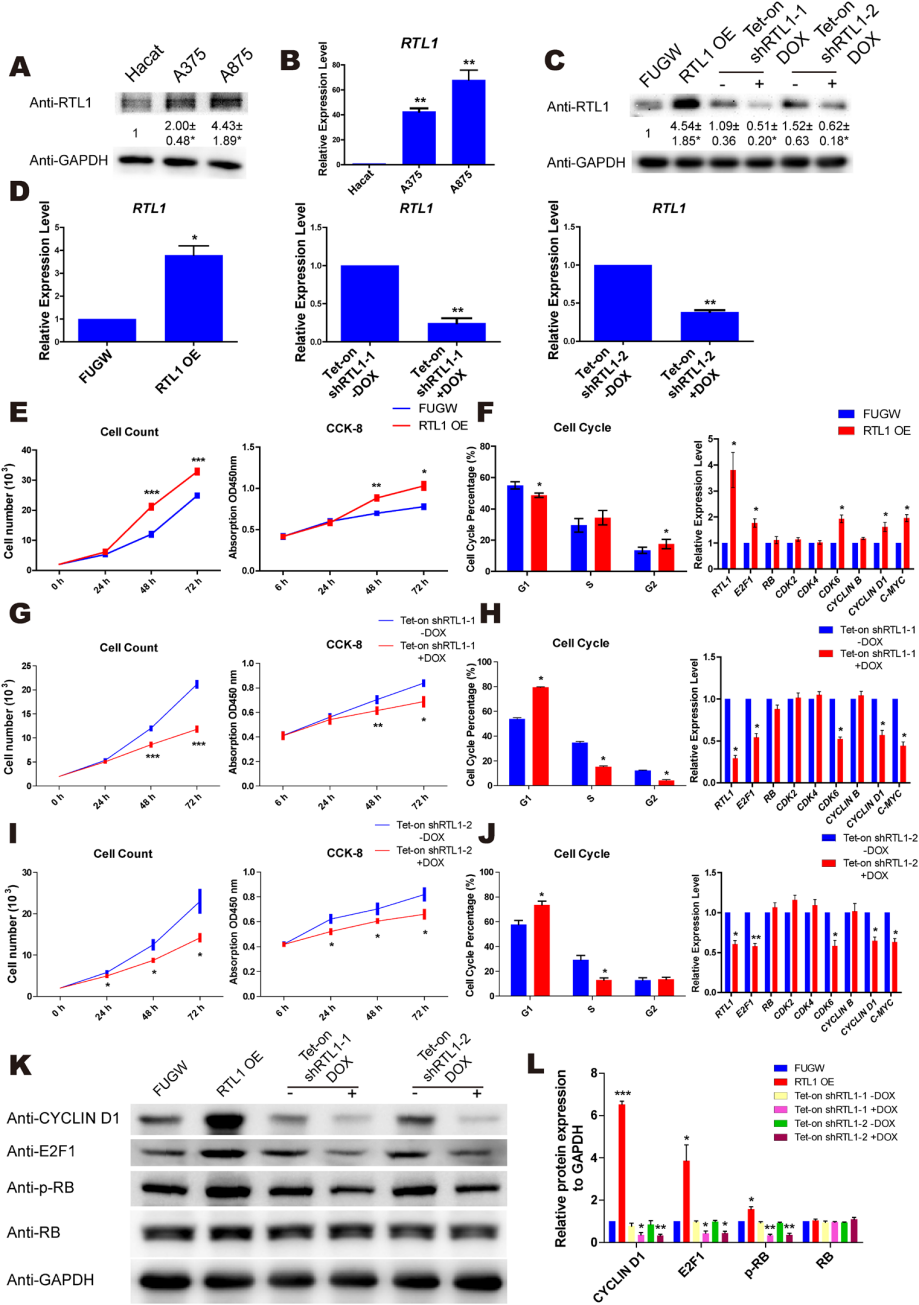
RTL1 promotes the proliferation of skin melanoma cells **(A)** Protein level of RTL1 in Hacat, A375 and A875 cells. The protein level was analyzed by Densitometry, compared with the Hacat cells ^*^ p<0.05; **(B)** mRNA level of the RTL1 expression in Hacat, A375 and A875 cells, RTL1 expression was higher in two melanoma cells, ^**^ p<0.01; **(C-D)** western blot and QPCR assays for the overexpression and knockdown efficiency of RTL1; the protein level was analyzed by Densitometry, compared with control group ^*^ p<0.05 and ^**^ p<0.01; **(E)** CCK8 and cell count assay for cell proliferation of A375 cells with RTL1 overexpression. RTL1 accelerated cell proliferation; ^*^ p<0.05, ^**^ p<0.01 and ^***^ p<0.001; **(F)** Fluorescence Activating Cell Sorter (FACS) assay assessing the cell cycle phases of A375 cells with RTL1 overexpression ^*^ p<0.05. And QPCR assay detecting expression of cell cycle-related genes; the RTL1 affected the expression of E2F1, CDK6, CYCLIN D1 and c-MYC, ^*^ p<0.05; (**G** and **I**) CCK8 and cell count assay for cell proliferation of A375 cells with RTL1 knockdown, ^*^ p<0.05, ^**^ p<0.01 and ^***^ p<0.001; (**H** and **J**) FACS assay assessing the cell cycle phases of A375 cells with RTL1 knockdown, ^*^ p<0.05. And QPCR assay detecting expression of cell cycle-related genes; the RTL1 affected the expression of E2F1, CDK6, CYCLIN D1 and c-MYC, ^*^ p<0.05 and ^**^ p<0.01; **(K)** Western blot analysis of cell cycle-related proteins in A375 cells with RTL1 overexpression or knockdown; **(L)** the protein level in (K) was analyzed by Densitometry, compared with the control group (FUGW and Tet-on shRNA without DOX), ^*^ p<0.05, ^**^ p<0.01 and ^***^ p<0.001.

To examine the expression of RTL1 in melanoma cells, a keratinocyte line (HaCat) and two melanoma cell lines (A875 and A375) were used in the study. We found that both the protein and mRNA levels of RTL1 were significantly higher in melanoma cells than in HaCat cells (Figure 2A, 2B). To explore the exact function of RTL1 in melanoma, we generated lentiviral vectors containing the full-length cDNA and shRNAs targeting RTL1 to overexpress and knockdown RTL1, respectively. The efficiency was confirmed by QPCR and western blot (Figure 2C, 2D).

A cell counting kit (CCK-8) and flow cytometry assays were used to investigate the role of RTL1 in melanoma. The results showed that knockdown of RTL1 inhibited the proliferation of melanoma cells and arrested the cell cycle transition from the G1 to the S phase, whereas overexpression of RTL1 promoted cell proliferation with cell cycle acceleration in the G1 phase. Furthermore, the cell cycle related genes were detected, and the expression levels of E2F1, CYCLIN D1, cyclin-dependent kinase 6 (CDK6) and c-MYC were up-regulated with RTL1 overexpression, whereas the knockdown of RTL1 down-regulated these genes (Figure 2E–2J). Additionally, the protein expression of these genes was detected by western blot analysis (Figure 2K, 2L). The functional experimental results of RTL1 in A875 cells are shown in Supplementary Figure 1A-1F. These findings indicated that RTL1 plays a critical role in the proliferation of melanoma cells.

### RTL1 activates the Wnt/β-Catenin pathway in melanoma cells

**Figure 3:**
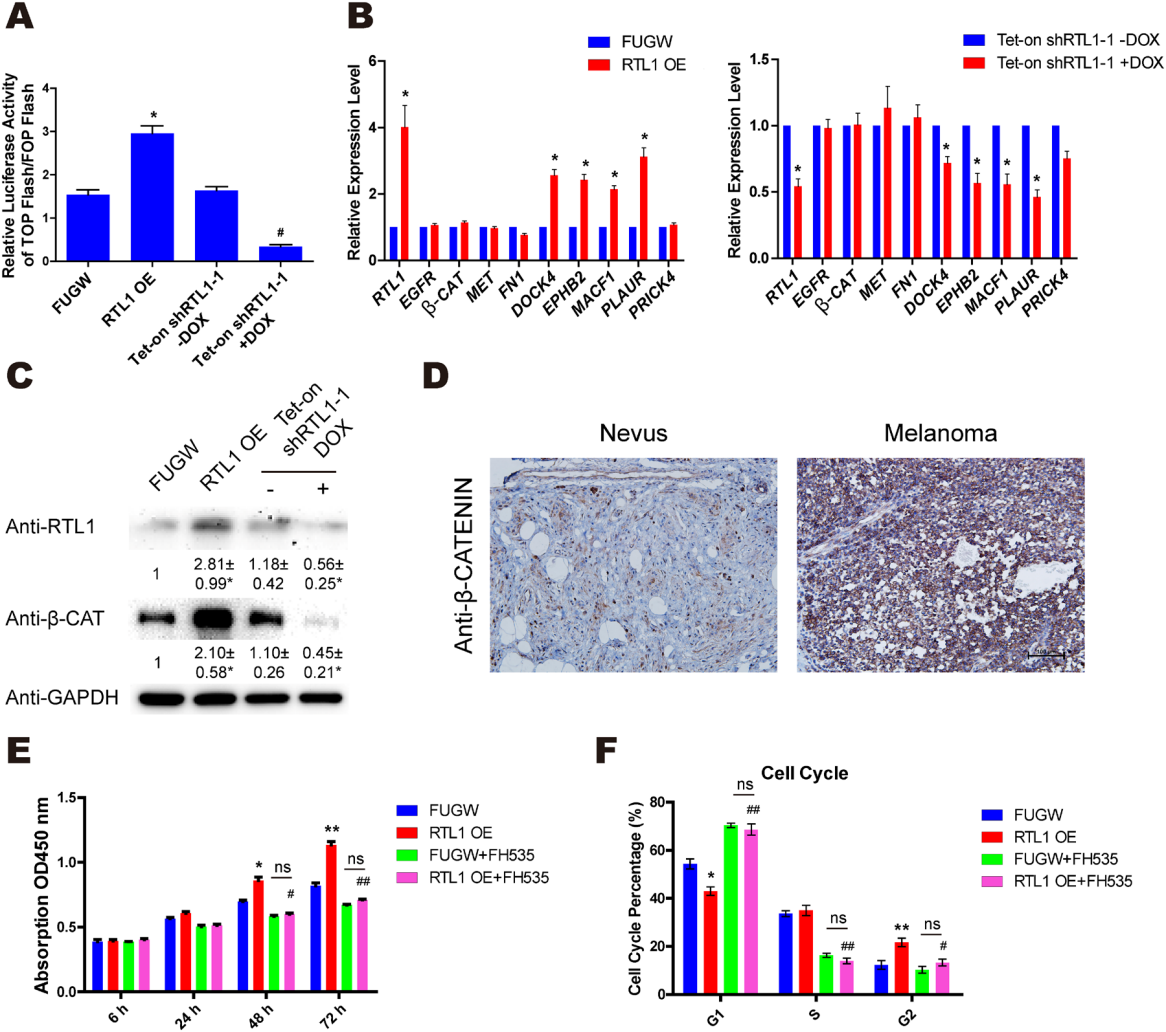
RTL1 activates the Wnt/β-Catenin pathway in melanoma cells **(A)** TOP flash and FOP flash relative luciferase active tests were used to detect the activity of the Wnt signalling with RTL1 overexpression or knockdown. Compared to FUGW, ^*^ p<0.05. Compared to Tet-on shRTL1-1 without DOX, ^#^ p<0.05; **(B)** the expression of β-Catenin stablizing genes in A375 cells with RTL1 overexpression or knockdown, ^*^ p<0.05; **(C)** β-Catenin expression in A375 cells with RTL1 overexpression or knockdown was detected by western blot; the protein level was analyzed by Densitometry, compared with the control group (FUGW and Tet-on shRNA without DOX), ^*^ p<0.05. **(D)** Analysis of the immunohistochemical expression levels of β-Catenin in human melanoma and nevus tissues, three tissues were examined; **(E)** CCK8 assay detecting the cell proliferation of A375 cells with RTL1 overexpression and with treatment with the WNT inhibitor FH535. Compared to FUGW, ^*^ p<0.05 and ^**^ p<0.01. Compared to RTL1-overexpressing cells, ^#^ p<0.05; **(F)** cell cycle of A375 cells with RTL1 overexpression and with treatment with the WNT inhibitor FH535 was detected by FACS. Compared to FUGW, ^*^ p<0.05 and ^**^ p<0.01. Compared to RTL1-overexpressing cells, ^#^ p<0.05 and ^##^ p<0.01.

The Wnt/β-Catenin pathway has been reported to be aberrantly activated in melanoma cells. To further investigate the functional mechanism of RTL1 in regulating cell proliferation in melanoma, we observed the effects of RTL1 on the activation of the Wnt/β-Catenin pathway by using TOP/FOP Flash luciferase reporter gene analysis. The results showed that overexpression of RTL1 enhanced the activation of Wnt signalling, whereas knockdown of RTL1 had the opposite effects (Figure 3A). However, the mRNA level of β-Catenin was not affected by modulating RTL1 expression. Furthermore, we observed the expression of β-Catenin and its stabilizing genes by QPCR, and we found that DOCK4, EPHB2, MACF1 and PLAUR were increased upon RTL1 overexpression and decreased with RTL1 knockdown (Figure 3B). Using western blot analysis, we found that the protein level of β-CATENIN was elevated after RTL1 overexpression, and it was decreased in the RTL1-knockdown cells (Figure 3C). We have also performed the corresponding experiments in lung cancer cell line (A549) and liver cancer cell line (HepG2), and confirmed the effects of RTL on Wnt signaling and β-Catenin stabilizing genes (Supplementary Figure 2A and 2B). Furthermore, we observed that the expression of β-CATENIN was significantly higher in human melanoma tissue than in the nevus by immunohistochemistry analysis (Figure 3D). The results of treatment with FH535, an inhibitor of Wnt signalling, showed that treatment of FH535 blocked the effects of RTL1 on cell proliferation and the cell cycle (Figure 3E, 3F), thus further confirming the critical role of RTL1 on Wnt signalling. These results suggested that RTL1 affects cell proliferation through regulating the Wnt/β-Catenin signalling pathway.

### β-Catenin is a crucial target of RTL1 in melanoma

**Figure 4:**
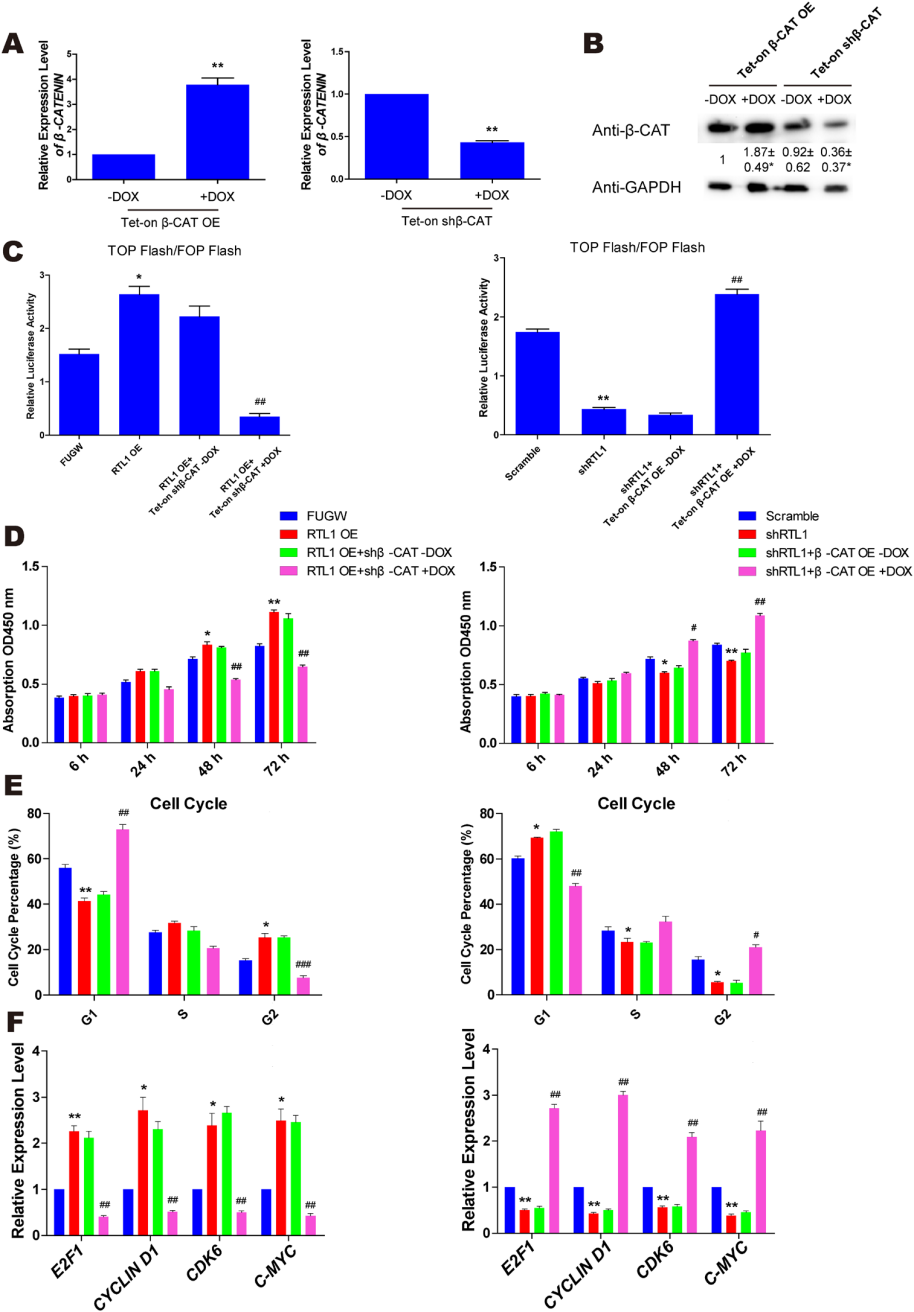
β-Catenin is the crucial target of RTL1 in melanoma **(A)** QPCR assay detecting the overexpression and knockdown efficiency of β-Catenin. Compared to the -DOX group, ^**^ p<0.01; **(B)** western blot assay detecting the overexpression and knockdown efficiency of β-Catenin; the protein level had been analyzed by Densitometry, compared with the control group (FUGW and Tet-on shRNA without DOX), ^*^ p<0.05. **(C)** The TOP flash and FOP flash relative luciferase activity assays were used to detect the activity of Wnt signalling in the RTL1-overexpressing cell line with β-Catenin knockdown, which was also performed in the RTL1-knockdown cell line with β-Catenin overexpression. Compared to FUGW or Scramble, ^*^ p<0.05 and ^**^ p<0.01. Compared to cells with RTL1 overexpression or knockdown, ^##^ p<0.01; **(D)** the cell proliferation assay for β-Catenin knockdown (left panel) or overexpression (right panel) in RTL1-overexpressing or RTL1-knockdown cells; **(E)** cell cycle assay for β-Catenin knockdown (left panel) or overexpression (right panel) in RTL1-overexpressing or RTL1-knockdown cells; **(F)** QPCR assay for the expression of cell cycle related genes of β-Catenin knockdown (left panel) or overexpression (right panel) in RTL1-overexpressing or RTL1-knockdown cells. Compared to FUGW or Scramble, ^*^ p<0.05 and ^**^ p<0.01. Compared to Tet-on β-Catenin knockdown or overexpression without DOX, ^#^ p<0.05 and ^##^ p<0.01.

To further understand the role of RTL1 in the Wnt/β-Catenin pathway, we generated a lentiviral vector containing the β-Catenin cDNA and shRNAs targeting the β-Catenin sequence *in vitro*. Lentiviral particles containing β-Catenin cDNA or shRNAs were used to infect melanoma cells. The overexpression and knockdown efficiency of β-Catenin was confirmed by QPCR and western blot analysis (Figure 4A, 4B). Using TOP/FOP Flash luciferase reporter gene analysis, we observed that in RTL1-overexpressing cells, knockdown of β-Catenin inhibited the activity of the Wnt pathway, whereas the overexpression of β-Catenin had a similar effect in RTL1-knockdown cells (Figure 4C). Moreover, overexpression of β-Catenin rescued the effect of RTL1 knockdown on cell proliferation, the cell cycle and the expression of cell cycle-related genes, whereas knockdown of β-Catenin reversed the promoting effect of RTL1 overexpression (Figure 4D–4F). These results suggested that RTL1 affects the Wnt signalling pathway by modulating β-Catenin.

### RTL1/β-Catenin engages in tumour growth *in vivo*

**Figure 5:**
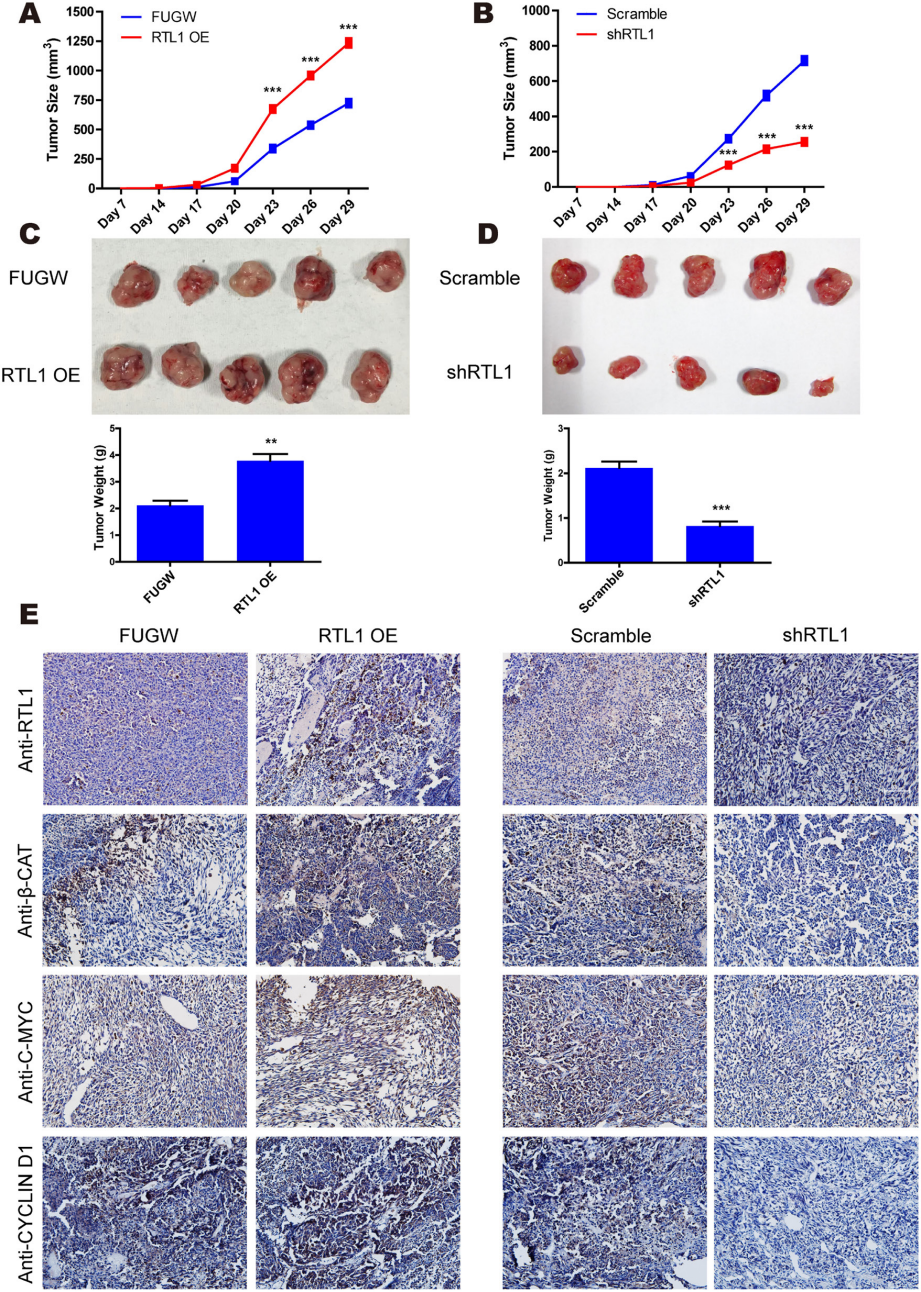
RTL1/β-Catenin engages in tumour growth *in vivo* **(A)** The tumour growth curve was obtained from RTL1-overexpressing (RTL1-OE) and control (FUGW) cells; **(B)** the tumour growth curve was obtained from RTL1-knockdown (shRTL1) and control (Scramble) cells; **(C)** images of tumours obtained from RTL1-overexpressing cells and the statistical analysis of tumour weight; **(D)** images of tumours obtained from RTL1-knockdown cells and the statistical analysis of the tumour weight. Compared to FUGW or Scramble, ^**^ p<0.01 and ^***^ p<0.001; **(E)** the immunohistochemical assay for the expression level of RTL1, β-CATENIN, c-MYC and CYCLIN D1 in tumour tissues.

To validate the effect of RTL1/β-Catenin on melanoma growth *in vivo*, nude mice were injected with RTL1-overexpressing or RTL1-knockdown melanoma cells. The tumour size was detected every 3 days, and the tumour weights were obtained at 4 weeks after injection. The results showed that RTL1 overexpression increased the tumour size and weight, whereas knockdown of RTL1 had the opposite effects (Figure 5A–5D). Furthermore, in an immunohistochemistry assay, we observed that the expression levels of RTL1, c-MYC, CYCLIN D1 and β-CATENIN in tumour tissues were increased after overexpression of RTL1 and decreased with RTL1 knockdown (Figure 5E), in agreement with previous findings in A375 cells. Therefore, β-Catenin and Wnt/β-Catenin signalling are the main downstream targets of RTL1 both *in vitro* and *in vivo*.

## DISCUSSION

Because melanoma is a highly aggressive skin cancer, the most effective period for curing melanoma is in early phases. Detection and control of melanoma in advance can greatly increase patient survival rate [31]. Intervening in melanoma proliferation is critical for the early treatment of melanoma. RTL1 is the key member of the *Dlk1-Dio3* imprinted region, and the loss of imprinting in this region and network disorder of imprint regulation are closely related to tumour formation [32]. Previous studies have shown that RTL1 is involved in several cancers, including lung cancer [12], squamous cell carcinoma of head and neck [13], and hepatocellular carcinoma [14–15], and the methylation level of RTL1 in melanoma patients is significantly lower than people without cancer [33]. In this study, we provided evidence that RTL1 is highly expressed in human melanoma tissues and that the positive rate in early and horizontal growth melanoma is much higher than in vertical growth and metastasis states. Moreover, RTL1 was found to promote the cell cycle transition from the G1 to the S phase and the proliferation of melanoma cells. These results suggested that RTL1 may be an important regulatory molecule in promoting early melanoma formation and development, and a potential predictor of melanoma.

β-Catenin deposits into the nucleus, binds to TCF/LEF-type transcription factors, and consequently activates downstream genes, such as Cyclin D1 and c-MYC [34]. In contrast, β-Catenin can be phosphorylated by casein kinase 1 and glycogen synthase kinase 3 in cytoplasm and subsequently degraded by the proteasome [35]. Abnormal activation of the Wnt/β-Catenin pathway has been reported as one of the predisposing factors in melanoma [19–22]. In the present study, we found that RTL1 overexpression activated the Wnt pathway, whereas knockdown of RTL1 inhibited the Wnt pathway. The inhibition of the nuclear accumulation of β-Catenin reversed the effects of RTL1 on melanoma cell proliferation, thus indicating that RTL1 promotes melanoma cell proliferation by modulating the Wnt/β-Catenin signalling pathway. Mechanistically, we found that RTL1 did not directly affect the expression of β-Catenin; instead, it increased the protein level of β-Catenin in melanoma cells by regulating the expression of DOCK4 and MACF1, both of which enhance the release of β-Catenin from the destruction complex and increase the stability of β-Catenin [36].

Immuno-suppression and resistance are the main factors that negatively influence targeted therapies for melanoma [29, 37]. Vemurafenib and Dabrafenib are two FDA-approved specific BRAF inhibitors and that are used to treat metastatic melanoma patients with BRAF mutations [38–40]. Although Vemurafenib rapidly improves the symptoms of melanoma patients, most patients acquire symptoms of drug resistance after 6-7 months [41–43]. Similar drug resistance has also been found in patients treated with an ERK inhibitor [44] or MEK inhibitor [45]. The occurrence of drug resistance in melanoma treatment has been found to be associated with multiple mechanisms, including secondary mutations, target gene mutations, changes in drug metabolism, and the activation of compensatory pathways [41]. Strikingly, Wnt/β-Catenin signalling appears to play a key role in immune resistance in melanoma. For instance, in melanoma with β-Catenin overexpression, T cells are prevented from entering the tumour tissue, and the infiltrated CD103^+^ dendritic cells are dramatically decreased [46]. Up-regulation of β-Catenin-LEF1 and concomitant down-regulation of YAP1 have been found to sensitize MAPKi-resistant melanoma [47]. In this study, we found that RTL1 activated the Wnt/β-Catenin pathway, thus suggesting that targeting RTL1 may provide a novel therapeutic target to melanoma patients or those with abnormally activated Wnt/β-Catenin signalling and related drug resistance.

In conclusion, we found that RTL1 is highly expressed in early rather than later stages of human melanoma tissues and that it critically regulates the cell cycle regulation and melanoma cell proliferation. Additionally, β-Catenin and Wnt/β-Catenin signalling are the main downstream targets of RTL1 both *in vitro* and *in vivo*. These findings indicate that RTL1 is involved in melanoma development and may serve as a potential new molecular target for melanoma diagnosis and therapeutics.

## MATERIALS AND METHODS

### Cells and patients

Two melanoma cell lines A375 (a human malignant melanoma cells) and A875 (a melanoma cell line), a lung cancer cell line (A549) and a liver cancer cell line (HepG2) were used in the study. Also a keratinocytes cell line HaCat had been used. All the cell lines were purchased from the Cell Bank at the Shanghai Institutes for Biological Sciences of the Chinese Academy of Sciences and cultured in Dulbecco's Modified Eagle's Medium (DMEM, GIBCO, USA) supplemented with 10% foetal calf serum, 100 U/ml streptomycin and 100 U/ml penicillin. A total of 32 human patients with melanoma, 4 patients with nevus, 4 patients with other skin cancer (two basal cell carcinoma and two squamous cell carcinoma), and 4 controls with normal skin were collected from Shanghai Skin Disease Hospital in China with written informed consent. This work has been approved by the ethical committees of our institution. The age of patients with melanoma are from 11 to 84, and the average age is 53.03±16.82. There was 16 female patients and 16 male patients. In 32 patients, there were 4 patients with early or superficial melanoma, 4 patients with horizontal growth melanoma, 12 patients with vertical growth melanoma and 12 with lymph nodes metastasis.

### Plasmid construction

The coding sequence of human wild-type RTL1 was cloned into the FUW lentivirus vector by AgeI and EcoRI restriction sites. The sequences of specific shRNAs targeting RTL1 and β-Catenin were: shRTL1-1, CTACCCAAGAATGTTCTATAA; shRTL1-2, CCGGAACTGTTTGACCAGTTA; shβ-Catenin, GTGCGCTCTTGAGGTTGTAAT, with CCTAAGGTTAAGTCGCCCTCG as scramble.

### Virus generation and infection

To generate lentiviruses, the plasmids carrying the specific sequences were transfected into HEK293T cells by the XtremeGENE HP DNA transfection reagent (Roche). After transfection for 48 h, the viruses were harvested. The cells were plated at a density of 1×10^5^ cells/well of 6-well plate and were infected with the viruses.

### Cell proliferation and cell cycle analysis

Cell proliferation was evaluated on the basis of using cell counts and a Cell Counting Kit-8 (CCK8, DOJINDO, Japan), as measured at 0 h or 6 h, 24 h, 48 h, and 72 h following plating. Data were analyzed according to the manufacturer's instructions.

The cell cycle was analyzed by FACS. The cells were harvested by trypsinization after cultured for 12 h, then fixed in 75% alcohol for 30 min. After washed 3 times by PBS, the cells were resuspended in the PBS, stained with PI for 15 min at room temperature in the dark, and then kept on ice until analyzed.

### Reverse transcription and quantitative real-time PCR

Total RNA was isolated from cells with TRIzol (Invitrogen, CA), and 500 ng of RNA was used to obtain cDNAs with the PrimeScript™ RT reagent Kit (TaKaRa, Japan). The cDNAs were used for real-time PCR analysis. The expression level of each gene was normalized to *GAPDH* and was further compared to the expression in the control group. The primers are shown in Supplementary Table 1.

### Western blot

Cells were lysed on ice for 25 min with radio-immunoprecipitation assay (RIPA) buffer (50 mM Tris, 0.1% sodium dodecyl sulphate, 1.0% IGEPAL CA-630, 150 mM NaCl, and 0.5% sodium deoxycholate, pH 8.0). The lyses were centrifuged at 12,000 g for 15 min at 4°C, and the protein quantity was determined using the bicinchoninic (BCA) method. Equal amounts of protein were loaded onto SDS-PAGE gels, separated by electrophoresis, transferred to membranes and incubated with the appropriate antibodies; then, the signals were developed on the basis of enhanced chemiluminescence (ECL) (1705061, Bio-Rad, USA). Primary antibodies were used in this study: RTL1 (ab107990, Abcam, UK), β-CATENIN (ab16051, Abcam, UK), E2F1 (sc-251, Santa Cruz, USA), c-MYC (ab32, Abcam, UK), CYCLIN D1 (sc-718, Santa Cruz, USA), and GAPDH (sc-47724, Santa Cruz, USA).

### Immunohistochemistry

Serial sections (10 μm) derived from formalin-fixed and paraffin-embedded blocks were dewaxed in xylene and rehydrated through graded ethanol to PBS. Antigen retrieval was achieved by heating sections in 10 mM citrate buffer (pH=6) before endogenous peroxidase blocking. Tissue sections were incubated with the following primary antibodies: β-CATENIN (1:300) (ab16051, Abcam, UK), RTL1 (1:100) (ab107990, Abcam, UK), c-MYC (1:200) (ab32, Abcam, UK), CYCLIN D1 (1:300) (sc-718, Santa Cruz, USA). Sections were then treated with secondary antibodies and counterstained with haematoxylin.

### Tissue microarray and evaluation of immunostaining

Melanoma cancer tissue microarrays were purchased from the SuperBiotek Company in Shanghai, China. Institutional Review Board permission for the use of samples was obtained. The evaluation of expression was simultaneously made by two blinded independent observers, and a consensus score was recorded, according to the staining intensity and the percentage of positive cells.

### Tumour xenografts in nude mice

The animal experiments were approved by the Institutional Animal Care and Use Committee of Tongji University and complied with all regulatory guidelines. Four-week-old nude mice were purchased from the National Resource Center for Rodent Laboratory Animals of China. Five mice were subcutaneously injected with 1×10^6^ cells of RTL1-overexpressing (RTL1-OE), RTL1-knockdown (shRTL1), or control (FUGW, scramble) cells. The tumours were monitored, and the volumes were calculated at 3-day intervals after appearance.

### Luciferase reporter gene assay

TOP flash and FOP flash constructs were used to evaluate β-catenin-dependent signaling events that drive the expression of TCF. TOP flash is a TCF reporter plasmid containing two sets of three copies of wild-type TCF binding sites driven by the thymidine kinase minimal prompter and upstream of a luciferase reporter gene. FOP flash contains mutated TCF binding sites driven by the same thymidine kinase promoter and also upstream of the same luciferase open reading frame as TOP flash. FOP flash was used as a negative control for TOP flash activity.

Cells were cultured in DMEM with 10% FBS and plated in 24-well plates at a density of 5×10^4^ cells per well. For each well, cells were transfected with either 0.5 μg of TOP flash or 0.5 μg of FOP flash reporter plasmid together with 0.1 μg of TK-Renilla luminescent reporter plasmid using Lipofectamine LTX and PLUS reagents (Invitrogen, CA). The assays were performed by the Dual-Luciferase Reporter Assay System (Promega, WI), and data were adjusted on the basis of the Renilla activity in the same sample. Each corrected TOP flash luciferase value was compared with the corresponding corrected FOP flash value. Three independent treatments were performed.

### Statistical analysis

The error bars represent the standard errors of the means (SEM) of three independent experiments. Data were tested for normalization and standard deviations to determine the appropriate statistical test (parametric versus non-parametric). Differences were considered statistically significant at ^*^p < 0.05, ^**^p < 0.01, and ^***^p < 0.001, or ^#^ p< 0.05, ^##^ p < 0.01, and ^###^ p < 0.001.

## SUPPLEMENTARY MATERIALS FIGURES AND TABLE




