# Supplementary material for: RTL1 promotes melanoma proliferation by regulating Wnt/β-catenin signalling
Source: Oncotarget. 2017 Nov 20;8(62):106026–37. doi: 10.18632/oncotarget.22523 (PMC5739699; doi:10.18632/oncotarget.22523)
Supplement: Supplementary file 1 [file oncotarget-08-106026-s001.pdf]

RTL1 promotes melanoma proliferation by regulating Wnt/β-catenin signalling

SUPPLEMENTARY MATERIALS

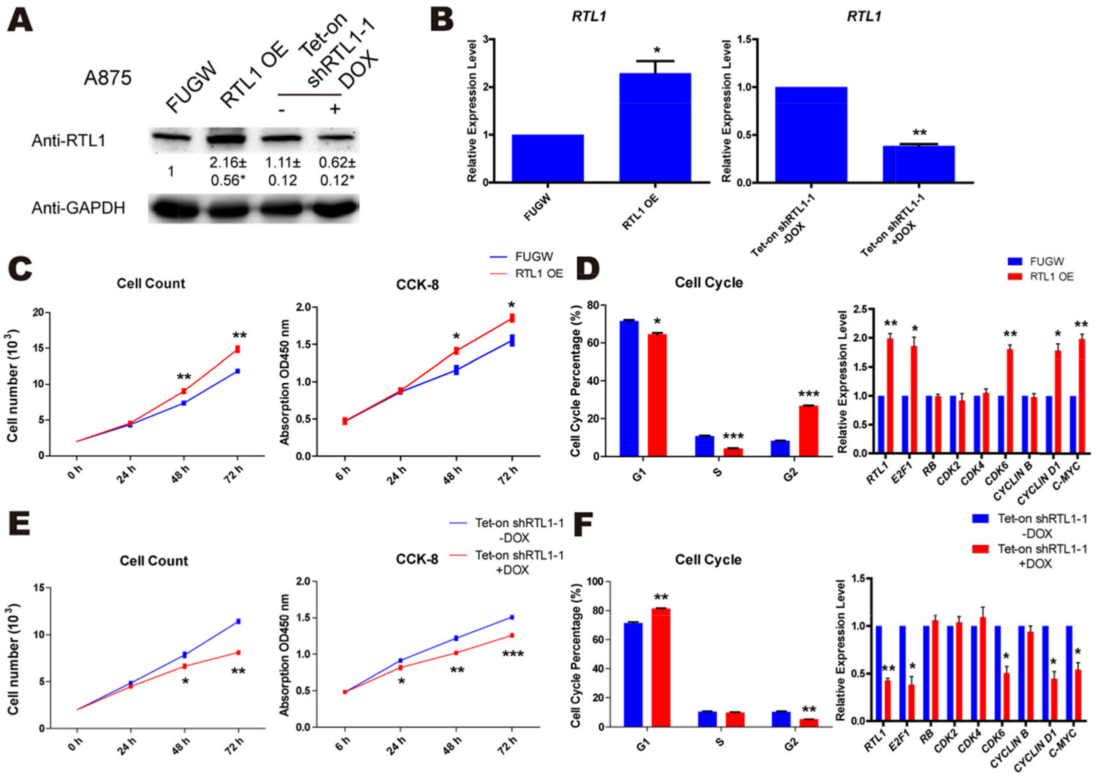

Supplementary Figure 1: RTL1 promotes the proliferation of skin melanoma A875 cells. **(A)** Western blot assay detecting the overexpression and knockdown efficiency of RTL1. The protein level also had been analyzed by Densitometry, compared with control group, * $p<0.05$. **(B)** QPCR assay detecting the overexpression and knockdown efficiency of RTL1, * $p<0.05$, ** $p<0.01$. **(C)** The cell count and CCK8 assay detecting cell proliferation of A875 cells with RTL1 overexpression. RTL1 accelerated cell proliferation, * $p<0.05$, ** $p<0.01$. **(D)** The FACS assay detecting the cell cycle phases of A875 cells with RTL1 overexpression and QPCR assay for cell cycle-related genes. * $p<0.05$, ** $p<0.01$, *** $p<0.001$. **(E)** The cell count and CCK8 assay for cell proliferation of A875 cells with RTL1 knockdown. * $p<0.05$, ** $p<0.01$, *** $p<0.001$. **(F)** The FACS assay for the cell cycle of A875 cells with RTL1 knockdown, and QPCR assay for cell cycle related genes. * $p<0.05$, ** $p<0.01$.

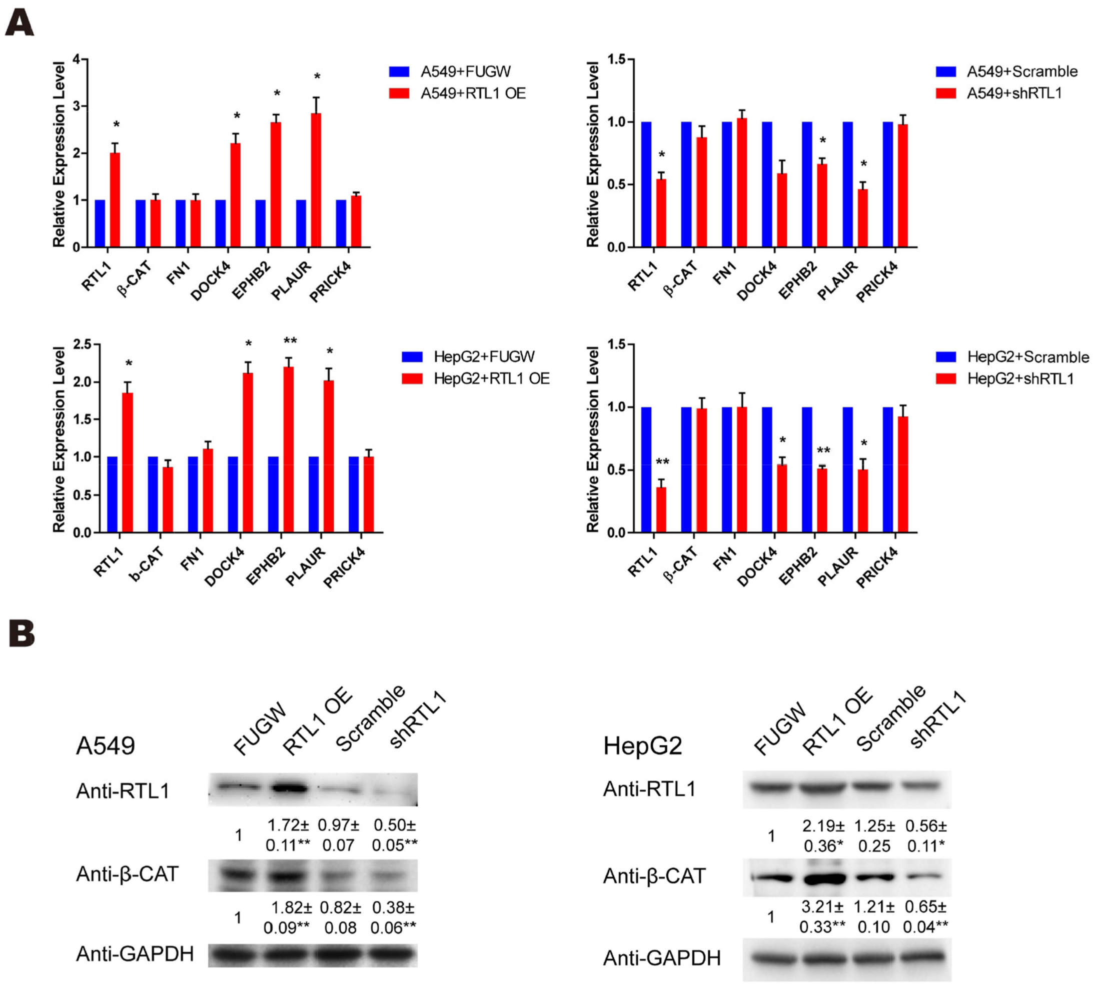

Supplementary Figure 2. RTL1 promotes the proliferation of lung cancer and liver cancer cells. (A) QPCR detecting the expression of β-Catenin stable related gene in A549 and HepG2 cells with RTL1 overexpression or knockdown, * $p<0.05$ and ** $p<0.01$; **(B)** β-Catenin expression in A549 cells and HepG2 cells with RTL1 overexpression or knockdown was detected by western blotting; the protein level had been analyzed by Densitometry, compared with the control group (FUGW and Scramble), * $p<0.05$ and ** $p<0.01$.

Supplementary Table 1: Primers for the quantitative RT-PCR

See Supplementary File 1
