# Supplementary material for: RTL1 promotes melanoma proliferation by regulating Wnt/β-catenin signalling
Source: Oncotarget. 2017 Nov 20;8(62):106026–37. doi: 10.18632/oncotarget.22523 (PMC5739699; doi:10.18632/oncotarget.22523)
Supplement: Supplementary file 2 [file oncotarget-08-106026-s002.docx]

**Supplemental Data**

**Table S1. Primers for the quantitative RT-PCR.**

| *RTL1-F* | GCTATGATTCAAACCCGGAGTT |
| --- | --- |
| *RTL1-R* | CCATGCTATAATCGGATGCCTC |
| *CYCLIN B- F* | TTGGGGACATTGGTAACAAAGTC |
| *CYCLIN B- R* | ATAGGCTCAGGCGAAAGTTTTT |
| *CYCLIN D-F* | CAATGACCCCGCACGATTTC |
| *CYCLIN D-R* | CATGGAGGGCGGATTGGAA |
| *E2F1-F* | ACGTGACGTGTCAGGACCT |
| *E2F1-R* | GATCGGGCCTTGTTTGCTCTT |
| *CDK4-F* | ATGGCTACCTCTCGATATGAGC |
| *CDK4-R* | CATTGGGGACTCTCACACTCT |
| *CDK6-F* | CCAGATGGCTCTAACCTCAGT |
| *CDK6-R* | AACTTCCACGAAAAAGAGGCTT |
| *C-MYC-F* | GTCAAGAGGCGAACACACAAC |
| *C-MYC-R* | TTGGACGGACAGGATGTATGC |
| *RB-F* | CTCTCGTCAGGCTTGAGTTTG |
| *RB-R* | GACATCTCATCTAGGTCAACTGC |
| *PRICK4-F* | GCTGCCTTGAGTGTGAAACG |
| *PRICK4-R* | TGCCCGTCATAGGTCATCTGT |
| *DOCK4-F* | CCTTCCAGCTACGTTCACTTG |
| *DOCK4-R* | GCCTTCATTACGCACATAGAGTT |
| *MACF1-F* | TCTCAGGCATCAAACTGCCC |
| *MACF1-R* | TTCACCTGTCGCTGCTTTAGG |
| *MET-F* | ACCATCTTTCGTTTCCTTTAGCC |
| *MET-R* | GGTTCACTGCATATTCTCCCC |
| *EGFR-F* | CCCACTCATGCTCTACAACCC |
| *EGFR-R* | TCGCACTTCTTACACTTGCGG |
| *EPHB2-F* | AGAAACGCTAATGGACTCCACT |
| *EPHB2-R* | GTGCGGATCGTGTTCATGTT |
| *FN1-F* | CGGTGGCTGTCAGTCAAAG |
| *FN1-R* | AAACCTCGGCTTCCTCCATAA |
| *PLAUR-F* | TGTAAGACCAACGGGGATTGC |
| *PLAUR-R* | AGCCAGTCCGATAGCTCAGG |
| *β-CAT-F* | CATCTACACAGTTTGATGCTGCT |
| *β-CAT-R* | GCAGTTTTGTCAGTTCAGGGA |
